# Sexual Ornaments, Body Morphology, and Swimming Performance in Naturally Hybridizing Swordtails (Teleostei: *Xiphophorus*)

**DOI:** 10.1371/journal.pone.0109025

**Published:** 2014-10-15

**Authors:** James B. Johnson, Danielle C. Macedo, Courtney N. Passow, Gil G. Rosenthal

**Affiliations:** 1 Department of Biology, Texas A&M University, College Station, Texas, United States of America; 2 Centro de Investigaciones Científicas de las Huastecas, Colonia Aguazarca, Calnali, Hidalgo, México; Arizona State University, United States of America

## Abstract

Determining the costs of sexual ornaments is complicated by the fact that ornaments are often integrated with other, non-sexual traits, making it difficult to dissect the effect of ornaments independent of other aspects of the phenotype. Hybridization can produce reduced phenotypic integration, allowing one to evaluate performance across a broad range of multivariate trait values. Here we assess the relationship between morphology and performance in the swordtails *Xiphophorus malinche* and *X. birchmanni*, two naturally-hybridizing fish species that differ extensively in non-sexual as well as sexual traits. We took advantage of novel trait variation in hybrids to determine if sexual ornaments incur a cost in terms of locomotor ability. For both fast-start and endurance swimming, hybrids performed at least as well as the two parental species. The sexually-dimorphic sword did not impair swimming performance *per se*. Rather, the sword negatively affected performance only when paired with a sub-optimal body shape. Studies seeking to quantify the costs of ornaments should consider that covariance with non-sexual traits may create the spurious appearance of costs.

## Introduction

A pervasive view in the study of sexual selection is that sexual ornaments are costly to the bearer, and that these costs are integral in conveying information to choosers as to the “quality” of the signaler [1]. Yet sexually-selected ornaments occur as part of a suite of traits that interact to determine performance and fitness [2]–[5]. Just as the benefits of sexual ornaments depend on their integration with other traits, so do their costs [2], [6]. In particular, exaggerated morphological structures like elongated feathers, fins, or horns are often assumed to impair locomotor performance; the evidence for such performance costs, however, is far from ubiquitous [2]. The lack of consensus likely emerges from the fact that it is ultimately the multivariate phenotype, including both sexually-selected and non-sexual traits, that affects locomotor performance [2], [3]. As a result, the interactions between sexual traits, non-sexual traits, and locomotor performance can be complex, and may result in the compensation of sexual ornament costs by non-sexual traits [2], [7]. Furthermore, this complexity could, in theory, result in exaggeration, rather than compensation, with sexual ornaments appearing more costly if paired with other traits which negatively influence locomotor performance.

To evaluate the fitness effects of sexually-selected traits in a multivariate context, we would ideally like to be able to decouple correlated traits to study an array of multivariate phenotypes in which traits vary independently. Arguably few natural phenomena can alter multivariate phenotypes as dramatically as hybridization [8], [9]. Hybridization can alter trait variances and covariances, thereby producing hybrid phenotypes that occupy a greater volume of phenotypic space relative to parental phenotypes [8]–[10]. This novel phenotypic variation can in turn affect ecologically relevant tasks such as locomotor performance [11]–[14]. We demonstrate that novel hybrid trait variation and its influence on locomotor performance can be used as a ‘natural laboratory’ [15] for evaluating functional relationships between traits and performance. For example, any assessment of the influence of a given trait on locomotor performance will be confounded by its co-expression with other trait values (i.e. trait correlations) [3], [16], [17]. Correlations among traits are often reduced in hybrids [9], [18], thus expanding the range of trait combinations in which to evaluate locomotor performance.

Here we take advantage of novel trait variation in natural hybrids of the swordtail fishes *Xiphophorus birchmanni* and *X. malinche* [19], [20] to evaluate the relationship between sexual ornaments, non-sexual traits and locomotor performance. *Xiphophorus malinche* males are similar to other swordtails and express an extension of the ventral rays of the caudal fin known as a sword [21]. *Xiphophorus birchmanni*, by contrast, have secondarily lost the sword; however, males bear an enlarged dorsal fin [21]. *Xiphophorus birchmanni* are also deeper-bodied, particularly in the mid-section, anterior region of the body, relative to *X. malinche*. Hybrids between the two species occupy a broad region of morphospace encompassing both parental species [19]. Thus, these fish differ radically with respect to not only sexual ornaments but also body morphology, which itself can have dramatic effects on swimming performance [22]–[25].

We evaluated the interaction between sexual ornaments, body shape and size on both unsteady swimming performance (fast-start velocity) and steady swimming performance (endurance swimming, time to fatigue). First, we determined if hybrids suffered reduced swimming performance relative to parentals. Second, we used novel phenotypic variation in a hybrid population to determine if body morphology is compensating for or exaggerating locomotor costs of sexual ornaments. Finally, we explored to what extent variation in hybrid performance is attributable to morphological and genetic similarities to parentals.

## Materials and Methods

### Specimen Collection

Animals were collected using funnel-traps between May–June 2010 from sites previously identified as *X. birchmanni*, *X. malinche* or hybrid [19], [20]. None of the sampled species are endangered or threatened. Collection permits we obtained from the Government of Mexico and were in hand at the time of collection. Parental males were collected from two *X. birchmanni* populations, Río Garces (20.94°, −98.282°; n = 15) and Río Coacuilco (21.099°, −98.587°; n = 7) and two *X. malinche* populations, Chicayotla (20.925°, −98.577°; n = 13) and Tlatzintla (20.881°, −98.799°; n = 6). The hybrid population Tlatemaco (21.023°, −98.79°; n = 35) was chosen because it is highly admixed, with sampled alleles in Hardy-Weinberg equilibrium and with no significant linkage disequilibrium among marker loci [20]. In addition, this hybrid population is morphologically intermediate to both parentals (JBJ and GGR, unpublished data; see Results). Furthermore, Tlatemaco displays reduced phenotypic integration (reduced trait correlations) between body shape, body size, sword length and dorsal fin size relative to parental populations. Specifically, the relative standard deviation of the eigenvalues, a measure phenotypic integration [26] was lowest in Tlatemaco hybrids (SD_rel_(*λ*) = 0.45) followed by *X. malinche* (SD_rel_(*λ*) = 0.59) and *X. birchmanni* (SD_rel_(*λ*) = 0.79). Thus, this hybrid population provides a broad range of trait values to evaluate function relative to parental populations. Animals were transported to our laboratory facilities at Texas A&M University in College Station, Texas, USA. Individuals were housed by population in 76-l aquaria and maintained in the lab for three weeks on a 12∶12 light cycle and on a diet of algae flake, decapsulated *Artemia* eggs, and bloodworms (*Glycera sp.*) prior to the start of swimming performance trials. Males were housed individually in 13-l aquaria between trials to track individual identity. Males were food-deprived for 48 hrs prior to the start of swimming performance trials to ensure they were in a post-absorptive state [27]. Research on animals was approved by the Institutional Animal Care and Use Committee of Texas A&M University and great care was taken to minimize animal suffering.

### Fast-start swimming performance trials

Fast-start swimming performance trials were conducted by placing an individual fish in a 16.21 cm×65 cm arena filled with filtered tap water to a depth of 4 cm. The test arena was illuminated using compact fluorescent lamps to minimize heating of the test arena. Water temperature was maintained at 21±0.2°C. Each fish was allowed 5 min to acclimate to the tank before the trial began. Fast-start behavior, i.e. c-starts [25] were elicited by startling the fish by striking the bottom of the test arena within 2 cm of the fish with one end of a wooden dowel (6.4 mm diameter) [28]. A high speed video camera (Casio Exilim Pro EX-F1, Casio Computer Co., Tokyo, Japan) recorded each fast-start event at 300 frames/s.

We measured fast-start velocity (*v*_max_), on a standardized location on the fish’s body, as follows. A line was fitted along the dorsal midsection of the fish in each frame starting at the tip of the snout to the end of the caudal peduncle. The point on the line which corresponded to half the standard length was used as a standard point for digitizing. Each video was digitized starting one frame before the fish’s movement began to the 15^th^ frame following movement (i.e. 53 ms of video was analyzed). Digitizing error was minimized using a mean-square quintic spline [29] executed in MATLAB 9.0 (The MathWorks, Natick, MA USA) [30].

### Steady swimming performance trials

We performed endurance swimming performance (i.e. time to fatigue) trials using a Brett type swim tunnel [31], [32]. The apparatus consisted of a flow-through tunnel with a test section (length = 45.7 cm, depth = 7.6 cm, width = 7.6 cm) with a matrix of plastic drinking straws upstream in order to minimize turbulent flow and a downstream grate to prevent the fish from leaving the test chamber. The apparatus was submerged in a 284-l aquarium and powered by a Leader Provort 540a propeller pump (Ladson SC, USA). Fish were tested using a modification of the protocol used by Royle et al. [33]. Water temperature was maintained at 21±1°C. Fish were placed individually in the test section and given 5 min to acclimatize. At the conclusion of the 5 min, flow was slowly increased to 20 cm/s for 5 min then increased to 30 cm/s for 1 min. Once the 30 cm/s interval was completed the flow was slowly increased to the test flow of 45 cm/s (∼10.8 body lengths/s). Exhaustion was defined following the criterion used in other studies of steady swimming performance [33]–[35]. If a fish stopped swimming during the 45 cm/s test period, the fish would end up being impinged against the back grating that covers the water outflow channel. If the fish remained pinned for 5 s the test chamber was tapped once, and if the fish did not resume swimming, the trial was ended and the time recorded. If the fish failed to exhaust by 25 min, the trial was terminated and the exhaustion time was recorded as 1500 s. The 25 min cutoff was justified by a preliminary dataset (n = 61) using a separate sample of fish. Of the fish in this dataset which swam for 25 min (n = 13), 92% continued to swim for >60 min.

Trial order was randomized but to assure our results are not biased by order we performed two generalized linear models (GLMs) with either fast-start swimming performance (velocity) or endurance swimming performance (time to fatigue) as dependent on which trial (fast-start or endurance trial) was first. Order of trial had no effect on swimming performance (GLM; fast-start: *F*_1, 74_ = 0.02, *P* = 0.887; endurance: *F*_1, 75_ = 0.15, *P* = 0.695).

### Morphometrics

**Figure 1:**
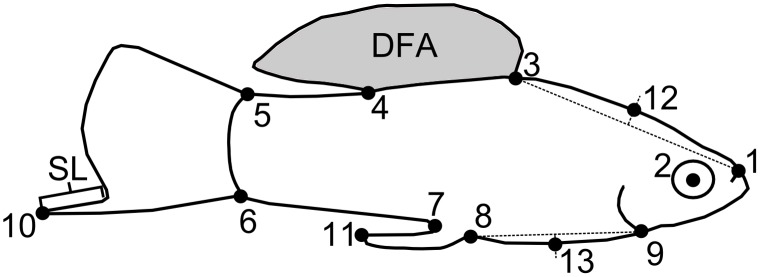
Landmark locations and measurements of sword length (SL) and dorsal fin area (DFA, shaded area).

At the conclusion of both swimming performance trials, fish were anesthetized using tricaine methanesulfate (MS-222). We took a lateral image of the right side of the body using a Nikon D90 digital camera with a 50 mm Nikkor lens (Nikon, Tokyo, Japan) mounted to a copy stand, and removed a small portion of the upper caudal fin for genetic analysis (see below). From each image, 13 landmarks were digitized using tpsDig (Figure 1) [36]. Landmarks included (1) upper lip, (2) eye, (3) anterior insertion of the dorsal fin, (4) posterior insertion of the dorsal fin, (5) dorsal insertion of the caudal fin ray, (6) ventral insertion of the caudal fin ray, (7) posterior insertion of the gonopodium, or intromittent organ (8) anterior insertion of the gonopodium and (9) the ventral occlusion of the operculum cover (Figure 1). The sword (10) and gonopodium tip (11) landmarks were subject to idiosyncratic differences in orientation in the images, i.e. position of the sword or gonopodium in a given image. These differences were removed by rotating both the sword and gonopodium tip (landmarks 10 and 11, respectively) to 45° relative to the centerline of the body [37]. Semi-landmarks for the nuchal hump (12) and belly (13) were interpolated from right angles from half the distance (shown as dotted lines) between landmarks 1 and 3 for landmark 12 and 8 and 9 for landmark 13, respectively. These semi-landmarks were taken into account during landmark alignment [38], [39]. Landmark coordinates were then subjected to generalized Procrustes superimposition where coordinates were translated, scaled and rotated, i.e. aligned [38], [39]. From the aligned coordinates, we calculated partial warps and uniform components (i.e. the weight matrix) which describe localized shape variation and uniform shearing in the X and Y dimensions, respectively [39], [40]. A body size statistic, centroid size, was also calculated as the square root of the sum squared distances for each individual’s landmark configuration to its centroid [39]. Alignment, calculation of the weight matrix and centroid size were performed using tpsRelw [41]. We also measured standard length (mm), sword extension length (mm) and dorsal fin surface area (mm^2^, Figure 1). Centroid size, sword length and dorsal fin area were log transformed.

### Genetic analysis

To determine the genetic composition (*X. malinche* vs. *X. birchmanni*) of each hybrid individual we genotyped hybrid males after Culumber et al. [20] using one mitochondrial marker and three unlinked intron SNPs. Each hybrid male was assigned a hybrid score based on the number of *X. malinche* alleles it bore at the four marker loci, ranging from zero to seven (one allele at the mitochondrial marker and two at each of the nuclear markers). Based on allele frequencies sampled in 2007, and given that these are physically unlinked markers, the probabilities that a fish assigned a hybrid index of 7 (i.e. pure *X. malinche*) or 0 (pure *X. birchmanni*) is in fact a hybrid are 0.12 and 6×10^−8^ respectively. Thus, we believe that our marker-based methods provide a reasonable index of hybridization.

### Statistical analysis

We used as series of generalized linear models (GLMs) to compare performance between species-types, order effects, hybrid index and the relationship between morphology and performance in hybrids. For models where the dependent variable was fast-start velocity, the error distribution was specified as Gaussian, whereas for models evaluating time to fatigue, were specified as a gamma error distribution. For all models outliers and leverage points were evaluated by eye using R statistical software [42].

Swimming performance is frequently expressed in units of body lengths (e.g. BL/s) in an effort to compare across animals of varying sizes. We did not perform size correction because when comparing species and populations we were explicitly interested in absolute differences in swimming performance, including body size. Further, we wished to evaluate the relationship between swimming performance and morphology, including size. We compared swimming performance among hybrids and parentals using two GLMs where swimming performance (either fast-start velocity or fatigue time) was dependent on species-type (*X. malinche*, *X. birchmanni* or hybrid). Differences between species-types were evaluated using Tukey’s HSD. To account for population effects, we also included population nested in species-type. We also evaluated the relationship between both fast-start velocity and endurance using Kendall’s rank correlation.

We evaluated the effect of hybrid morphological traits (sexual ornaments, body shape and size) on swimming performance (either fast-start velocity or time to fatigue) with model selection [43] using the glmulti package [44] in the R statistical software platform [42]. Because we were interested in body shape *per se*, we excluded landmarks 10 (sword tip) and 11 (gonopodium; Figure 1); otherwise, alignment and calculation of partial warps and uniform components were as presented above (see *Morphometrics*). Partial warps and uniform components were subjected to PCA to reduce dimensionality [39], [45]. The first three principal components accounted for 22%, 19% and 14% of the variance in hybrid body shape, respectively, and were retained for further analysis. All possible models were considered, ranging from the full model (dorsal fin area, sword length, body shape PC 1 (BSPC1), PC 2 (BSPC2), PC 3 (BSPC3) and log centroid size and all pairwise interaction terms) to the null model (intercept only). If interaction terms were retained their respective main effects were likewise retained, i.e. the principle of marginality was observed [44]. Models were evaluated using Akaike Information Criteria (finite sample correction, AICc) [43]. In addition, we report the difference between the AIC_C_ score of a given model and the lowest AIC_C_ score (i.e. ΔAIC_C_). Models which differ within 2 AIC_C_ units from the model with the lowest AIC_C_ (ΔAIC_C_<2) are considered equally supported [46]. We also evaluated relative importance of main effects and interaction terms individually using the sum of the relative evidence weights for each model in which a given term appears, terms which exceed an importance value of 0.8 were considered important [44], [47]. If sexual ornaments are costly *per se*, we expected that a negative relationship between ornament and locomotor performance would be retained in well supported models and will show greater relative importance. Furthermore, significant interaction terms between body shape and ornaments would suggest that ornaments are being compensated for or exaggerated by body shape and are not costly *per se*. Significant interaction terms were visualized using non-parametric thin-plate spline regression to create a performance surface [48], [49]. Estimation of performance surfaces was performed in R [42] using the fields package [50] (smoothing parameter lambda = 0.001).

To determine if observed differences in morphology and swimming performance between parentals were mirrored by variation among hybrids, we evaluated vectors describing morphological variation between both parental species, and between hybrids that differed in performance. Specifically, we tested whether morphological differences between hybrids that did and did not exhaust in the endurance swimming trials were consistent with morphological differences between *X. malinche* and *X. birchmanni.* We performed this analysis only for the endurance data, since parentals and hybrids only marginally differed in fast-start performance (see Results). If the relationship between morphology and endurance in hybrids mirrors species differences (e.g. hybrids that did not exhaust are more morphologically similar to their better performing parental) we *a priori* expect the orientation between both vectors to be parallel. We included both the body and the sword ornament (landmark 10) and the gonopodium (landmark 11), which has been shown to influence swimming performance in other poeciliids [51]. Partial warps and uniform components were size-adjusted by taking residuals in a MANOVA model where the partial warp and uniform components were dependent on log centroid size (*F*_22, 54_ = 6.07, *P* = <0.001). Means of each size-adjusted partial warp and uniform component were calculated for *X. birchmanni*, *X. malinche*, hybrids that exhausted and hybrids that did not exhaust, and evaluated using MANOVA [52]. Using these means, two vectors were created: the first described variation between the parental species, *X. birchmanni* and *X. malinche* and the second described variation between hybrids that exhausted and hybrids that did not exhaust following the methods described in Collyer and Adams [52]. We determined if the two vectors were oriented similarly by calculating the angle between them [52]. Permutation tests (1000 iterations) were used to evaluate the significance of the observed angle between vectors using a residual randomization approach [52]. *P*-values were calculated to evaluate the null hypotheses that the two vectors are parallel [52]. Analysis of phenotypic vectors was performed in R [42] using a modification of the script provided in Collyer and Adams [52]. To visualize the differences between vectors we subjected the partial warps and uniform components to PCA [39], [45], [52]. The first two PC scores explained 75% and 7% of the variance respectively. Both PC1 and PC2 were size-adjusted by taking residuals using MANOVA (*F*_2, 74_ = 30.98, *P* = <0.001). Morphological vectors were visualized by plotting means and standard error of residual PC 1 and PC 2 scores for *X. birchmanni* and *X. malinche*, hybrids that exhausted, and hybrids that did not exhaust. These means were used to create vectors describing morphological change between parentals and hybrids in residual PC space. In addition, we visualized a performance surface describing variation in performance in morphological space using non-parametric thin-plate spline regression (see above). The performance surface is provided for heuristic purposes and is independent of the calculation of the means, standard errors, vectors and contrasts.

To evaluate the relationship between fast-start swimming performance and genetic similarity of hybrids to parental species, we performed two GLMs where swimming performance (either fast-start velocity or fatigue time) was dependent on hybrid index.

## Results

### Parentals and hybrid swimming performance

**Figure 2:**
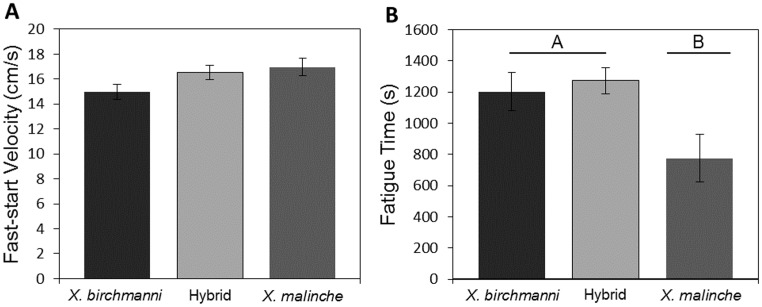
Means and standard errors for fast-start velocity (A) and time to fatigue (B) by species-type. Letters and lines indicate similarities between means determined using Tukey’s HSD.

Fast-start velocity was marginally significantly different among species-types (*X. birchmanni, X. malinche,* or hybrids; *F*_2, 71_ = 2.8, *P* = 0.068). *Xiphophorus birchmanni* were slowest (mean = 14.95 cm/s, SE = 0.59), *X. malinche* fastest (mean 16.97 cm/s, SE = 0.69) and hybrids intermediate (mean = 16.53 cm/s, SE = 0.55) in fast-start velocity (Figure 2A). Population nested in species-type was also marginally significant (*F*_2, 71_ = 2.74, *P* = 0.071).

We found significant variation between parentals and hybrids with respect to endurance swimming performance (*F*_2, 70_ = 6.41, *P* = 0.003). Hybrids showed the greatest endurance (mean fatigue time = 1273.17 s, SE = 85.79) and *X. malinche* the least (mean fatigue time = 776.28 s, SE = 153.86), with *X. birchmanni* intermediate in endurance (mean fatigue time = 1203.64 s, SE = 119.82; Figure 2B). Tukey’s HSD post-hoc analysis suggests that fatigue time was not significantly different between hybrids and *X. birchmanni* (z = −0.371, *P* = 0.925) but that both hybrids (z = 2.74, *P* = 0.016) and *X. birchmanni* (z = 2.354, *P* = 0.047) had significantly greater endurance than *X. malinche*. Population nested in species-type was also significant (*F*_2, 70_ = 31.41, *P* = <0.001).

There was no detectable relationship between time to fatigue and fast-start velocity (Kendall’s rank correlation, τ = −0.12, z = −1.33, *P* = 0.183).

### Hybrid morphology and performance

**Figure 3:**
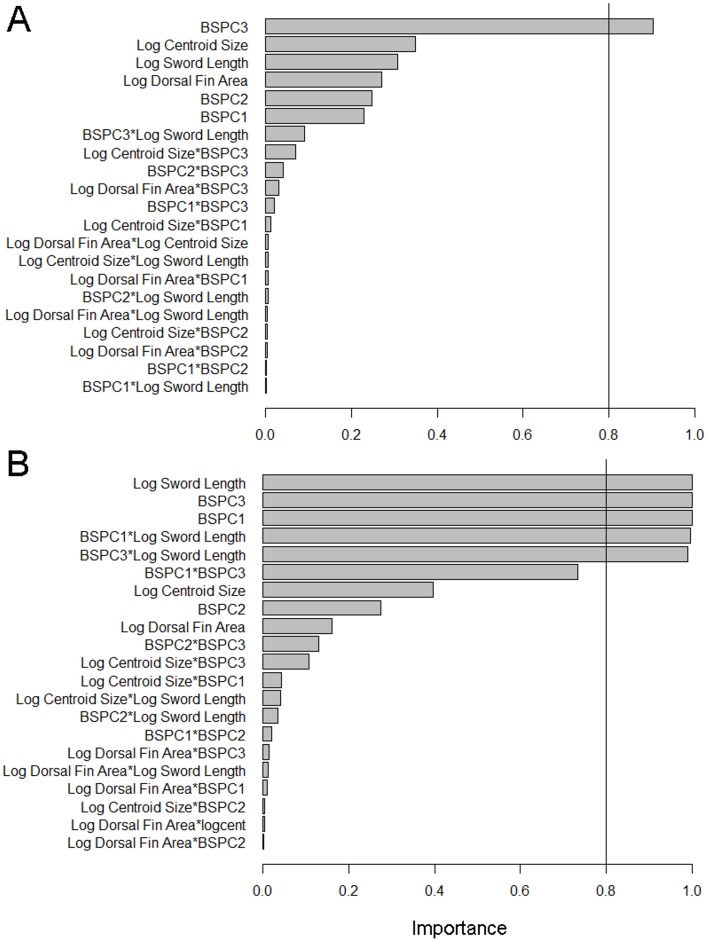
Importance of morphological variables in predicting fast-start velocity (A) and time to fatigue (B) in hybrids.

**Figure 4:**
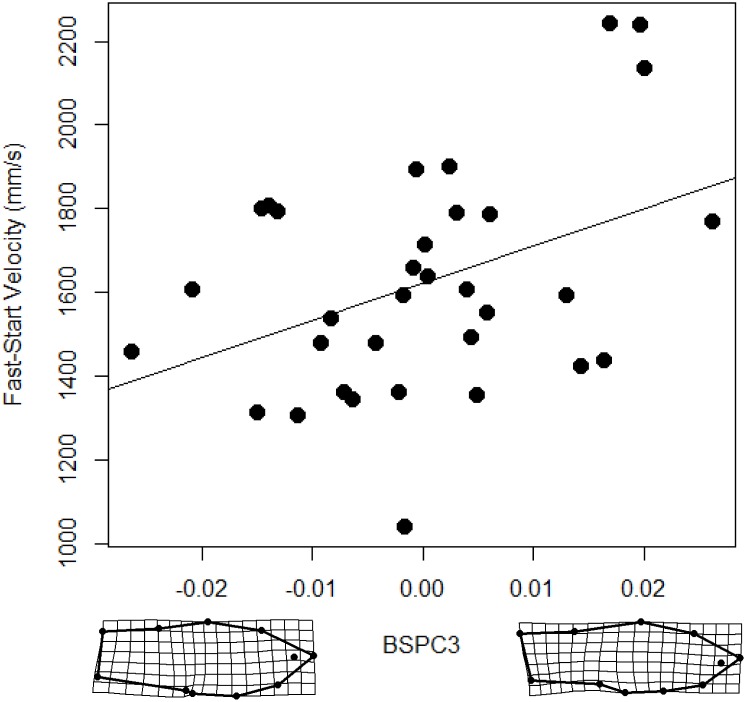
The relationship between body shape PC 3 (BSPC3) and fast-start velocity in hybrids.

Model selection indicated that morphology had little association with fast-start swimming performance in hybrids. The model with the lowest AIC_c_ score consisted of only a minor component of body shape, body shape PC 3 (Table 1). Among other equally supported models (i.e. ΔAIC_c_<2; Table 1) body shape PC 3 occurred in all models and was the only term which was statistically significant (Table 1). Furthermore, only body shape PC 3 exceeded the 0.8 threshold of relative importance (Figure 3A). Fish which were fastest were those with elongated midsections (Figure 4).

**Table 1:** Results for the best supported models (ΔAIC_c_<2) predicting fast-start velocity.

Model	AIC_c_	ΔAIC_c_	Effects	SS	DF	F	P
1	463.37	0	Body Shape PC 3	385834	1	6.07	**0.019**
			Error	1969327	31		
2	464.83	1.46	Body Shape PC 3	368464	1	5.81	**0.022**
			Log Centroid Size	67053	1	1.06	0.312
			Error	1902274	30		
3	464.94	1.57	Body Shape PC 3	424214	1	6.67	**0.015**
			Log Dorsal Fin Area	60377	1	0.95	0.338
			Error	1908949	30		
4	465.36	1.99	Body Shape PC 3	396099	1	6.15	**0.019**
			Log Sword Length	36223	1	0.56	0.459
			Error	1933104	30		

**Figure 5:**
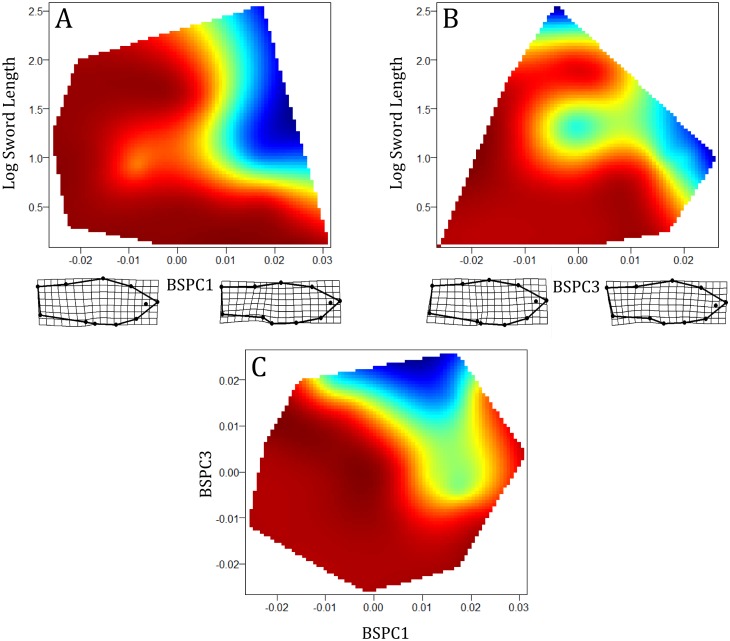
Performance landscapes for the significant interaction effects of log sword length by body shape PC 1 (BSPC1; A), log sword length by body shape PC 3 (BSPC3; B) and body shape PC 1 body shape by body shape PC 3 (C). Red represents longer endurance time.

Unlike fast-start performance, endurance swimming performance in hybrids was strongly influenced by morphology. Only one model was supported (i.e. ΔAIC_c_<2; Table 2) and consisted of body shape PC 1, body shape PC 3, sword length, and the interaction terms of body shape PC1 by body shape PC 3, log sword length by body shape PC 1 and finally, log sword length by body shape PC 3 (Table 2). Interaction terms of all morphological traits were significantly related to time to fatigue (Table 2). In addition, terms with importance values which exceeded the 0.8 threshold were body shape PC 1, body shape PC 3, log sword length and the interaction terms of body shape PC1 by sword length and body shape PC 3 by log sword length (Figure 3B). Surface visualizations of the significant interaction terms of body shape (PC 1 and PC 3) and sword length show that sword length had a negative effect on endurance swimming only when paired with a small anterior body shape (body shape PC 1) and elongated mid-section (body shape PC 3; Figure 5).

**Table 2:** Results for the best supported model (ΔAIC_c_<2) predicting time to fatigue.

Model	AIC_c_	ΔAIC_c_	Effects	SS	DF	F	P
1	486.51	0	Body Shape PC 1	0.18	1	1.06	0.313
			Body Shape PC 3	0.72	1	4.23	0.050
			Log Sword Length	3.25	1	19.08	**0.000**
			Body Shape PC 1*Body Shape PC 3	1.25	1	7.35	**0.012**
			Body Shape PC 1*Log Sword Length	2.58	1	15.14	**0.001**
			Body Shape PC 3*Log Sword Length	1.85	1	10.85	**0.003**
			Error	4.61	27		

### Parental and hybrid phenotypic and performance trajectories

**Figure 6:**
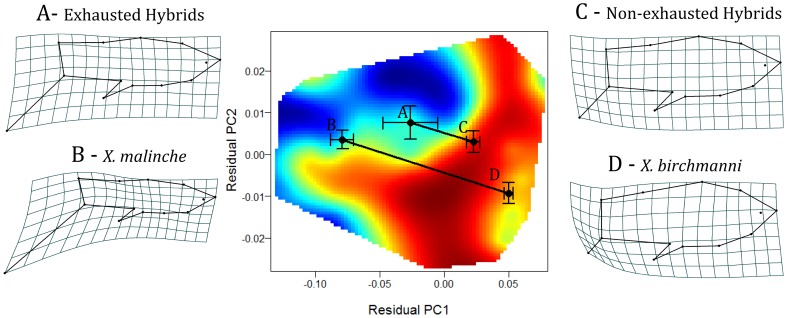
Performance landscape showing variation in morphology and swimming performance between parentals (*X. malinche* and *X. birchmanni*) and between hybrids that exhausted and hybrids that did not. Red represents longer endurance time. Means and standard errors of residual PC 1 and residual PC 2 for hybrids (A and C) and parentals (B and D) are shown. Morphometric visualizations were magnified by a factor of 3 [83]–[85].

MANOVA indicated significant differences in morphology between parental species and between exhausting and non-exhausting hybrids (*F*_66, 156.14_ = 4.89, *P* = <0.001). Vectors describing morphological variation between parental species and between exhausting and non-exhausting hybrids were parallel in orientation (θ = 20.8°, *P_rand_* = 0.99) and differed in length (*D*_parental_ = 0.13, *D*_hybrid_ = 0.05, *P*_rand_ = 0.001). Hybrid fish that did not exhaust were predominantly *X. birchmanni*-like morphologically. These fish had, on average, deeper, more anterior-allocated bodies, shorter, tapered caudal peduncles, larger dorsal fins and very short or absent swords (Figure 6).

### Genetic association with swimming performance in hybrids

Genetic similarity to the two parent species did not predict performance. There was no association between hybrid index for fast-start (*F*_1, 30_ = 0.72, *P* = 0.401) or endurance swimming performance (*F*_1, 28_ = 0.02, *P* = 0.89).

## Discussion

Sexual ornaments are thought to encode information pertaining to the “quality” of the bearer [53]–[56], but see [57]–[59]. A central pillar of this argument is that sexual ornaments are often costly to express [56], [60]. The honesty of sexual signals is then enforced by condition-dependence, whereby individuals in good “condition” are better equipped to bear these costs [61], [62]. However, as Dobzhansky [63] famously stated, “no trait is an island”. Interactions between sexual and non-sexual traits may alter the burden of sexual ornaments [2], [7]. Thus, to accurately evaluate the true costs of sexual ornaments we must consider how sexual and non-sexual traits interact to influence costs. Our data suggest that the apparent locomotor costs of the sword are a by-product of the sword being paired with a sub-optimal body shape. Fish that performed well in the endurance swimming predominantly had narrow, tapered caudal peduncle and increased anterior body depth (BSPC 1, Figure 5 and Figure 6) and reduced mid-sections (although this explained a rather small portion of the phenotypic variance, BSPC 3, Figure 5 and Figure 6). These results are congruent with theoretical and empirical findings describing the relationship between body morphology and steady swimming performance [22]. In short, a narrow, tapered caudal peduncle and increased anterior body depth, as found in *X. birchmanni* and *birchmanni-*like hybrids, should benefit endurance swimming performance by minimizing drag and maximizing thrust [22], [24]. In line with our findings, Kruesi and Alcaraz [31] found that *X. montezumae* males with more rotund bodies had greater swimming performance, and incurred relatively little change in swimming performance after sword removal. Furthermore, while we did find an association between a minor component of body shape (body shape PC 3) and fast-start velocity (Table 1, Figure 4 and Figure 5), we found no relationship between sexual ornaments and fast-start velocity (Table 1, Figure 4 and Figure 5). Fast-start velocity and time to fatigue represent measures of the two primary forms of fish swimming performance, unsteady and steady swimming performance, respectively [22], [24], [64]. We cannot exclude the possibility that sword and dorsal fin sexual ornaments are costly to an unmeasured aspect of performance. For example, the conspicuousness of the sword increases predation risk [65]–[67]. Furthermore, in *X. montezumae,* where the sword is exaggerated to the point that it exceeds body length, it increases metabolic demands [68]. However, our results strongly suggest that in this system, sexual ornaments by themselves incur little, if any, cost to swimming performance.

There have been mixed results on the relationship between swimming performance and the sword ornament in *Xiphophorus*: some studies have found a negative relationship between swimming performance and presence of the sword [31], others have found no relationship [34], [69]–[72], and one has even found positive effects of the sword on swimming performance [73]. This lack of consensus may be the result of several factors. For example, experimental removal of the sword [31], [69] can be informative [74] but may affect the relationship between other traits and swimming performance [70]. In addition, discrepancies among studies may be due to differing patterns of covariation between ornaments and non-sexual traits among species which, as our study suggests, can alter the locomotor costs of sexual ornaments [2], [6]. These potential problems have been avoided in our study by taking advantage of the natural and novel phenotypic variation created via hybridization and highlight the importance of evaluating both the costs and the benefits of sexually selected traits from a multivariate perspective.

Fish morphology should have a strong association with swimming performance [22], [24]. A wealth of empirical and theoretical work provides expectations with respect to both steady and unsteady swimming performance [22], [24], some, but not all, of which were supported by our data. Steady swimming performance should be greater in fish with narrow, tapered caudal peduncles and an anteriorly shifted center of mass [22], [24]. These morphological elements are seen in the robust body shapes of *X. birchmanni* and *X. birchmanni*-like hybrid males who, performed better in endurance swimming trials relative to *X. malinche* and *X. malinche*-like hybrids (Figure 5 and Figure 6). Conversely, a reduced anterior body and large caudal may increase fast-start velocity [22], [24]. We found a minor component of body shape (PC 3, 14%) was significantly related to fast-start velocity in hybrids (Table 1). PC 3 describes an increase in the body mid-section area (between the dorsal and anal fin insertions; Figure 4). This does not meet our expectations for morphological variation which would improve unsteady swimming performance. In addition, steady and unsteady swimming performance are expected to be negatively correlated as the morphological variation which maximizes one form of swimming is divergent [22], [24]. Our results do not support this expectation as the correlation between fast-start velocity and time to fatigue was not significant.

Locomotor performance has a profound impact on the fitness of animals. In fishes, fast-start swimming performance is associated with predator evasion [28], [75]–[77], whereas endurance swimming could influence competitive interactions, locating suitable micro-habitat, and dispersal [23], [78], [79]. Furthermore, improved or compromised locomotor performance may indicate differential vulnerability to predators or dispersal ability of hybrid populations over parentals [11], [13], [80]. We observed that hybrids performed as well if not slightly better than the best-performing parental species in *both* fast-start and endurance swimming performance (Figure 2). These findings are consistent with previous observations in the *birchmanni-malinche* hybrid system. For example, we observed that fish with a *X. birchmanni*-like body shape performed better in endurance swimming trials (Figure 5 and Figure 6). Previous work indicates that the *X. birchmanni*-like morphology is overrepresented in the hybrid zone [19]. Thus the prevalence of *X. birchmanni*-like morphology among hybrid populations may result from improved endurance swimming performance and thus dispersal ability of *X. birchmanni* like hybrids. Furthermore, hybrid male phenotypes are not costly with respect to sexual selection; indeed, females of both parental species fail to prefer conspecifics over hybrids in mate-choice trials, and express preferences for some hybrid phenotypes [81]. Thus, hybrids in this system do not appear to suffer costs in either attractiveness or locomotor performance, and may in fact have greater fitness at the intermediate elevations where they are found [82].
